# Sanguineous Pericardial Effusion and Cardiac Tamponade in the Setting of Graves' Disease: Report of a Case and Review of Previously Reported Cases

**DOI:** 10.1155/2016/9653412

**Published:** 2016-06-29

**Authors:** Peter V. Bui, Sonia N. Zaveri, J. Rush Pierce Jr.

**Affiliations:** Department of Internal Medicine, The University of New Mexico, Albuquerque, NM 87106, USA

## Abstract

*Introduction*. Pericardial effusion in the setting of hyperthyroidism is rare. We present a patient with Graves' disease who developed a sanguineous pericardial effusion and cardiac tamponade.* Case Description*. A 76-year-old man presenting with fatigue was diagnosed with Graves' disease and treated with methimazole. Two months later, he was hospitalized for uncontrolled atrial fibrillation. Electrocardiography showed diffuse low voltage and atrial fibrillation with rapid ventricular rate. Chest radiograph revealed an enlarged cardiac silhouette and left-sided pleural effusion. Thyroid stimulating hormone was undetectable, and free thyroxine was elevated. Diltiazem and heparin were started, and methimazole was increased. Transthoracic echocardiography revealed a large pericardial effusion with cardiac tamponade physiology. Pericardiocentesis obtained 1,050 mL of sanguineous fluid. The patient progressed to thyroid storm, treated with propylthiouracil, potassium iodine, hydrocortisone, and cholestyramine. Cultures and cytology of the pericardial fluid were negative. Thyroid hormone markers progressively normalized, and he improved clinically and was discharged.* Discussion*. We found 10 previously reported cases of pericardial effusions in the setting of hyperthyroidism. Heparin use may have contributed to the sanguineous nature of our patient's pericardial effusion, but other reported cases occurred without anticoagulation. Sanguineous and nonsanguineous pericardial effusions and cardiac tamponade may be due to hyperthyroidism.

## 1. Introduction

Hyperthyroidism is found in 1.3% of the United States population (12 years and older), and cardiac manifestations are common [1]. However, of those cardiac findings, pericardial effusion associated with hyperthyroidism has rarely been reported, limited to a small number of case reports primarily in the setting of Graves' disease [2–8]. We report a patient with Graves' disease and hyperthyroidism who developed a sanguineous pericardial effusion with cardiac tamponade. We reviewed the literature for previous case reports of patients with hyperthyroidism and pericardial effusion.

## 2. Case Description

**Figure 1:**
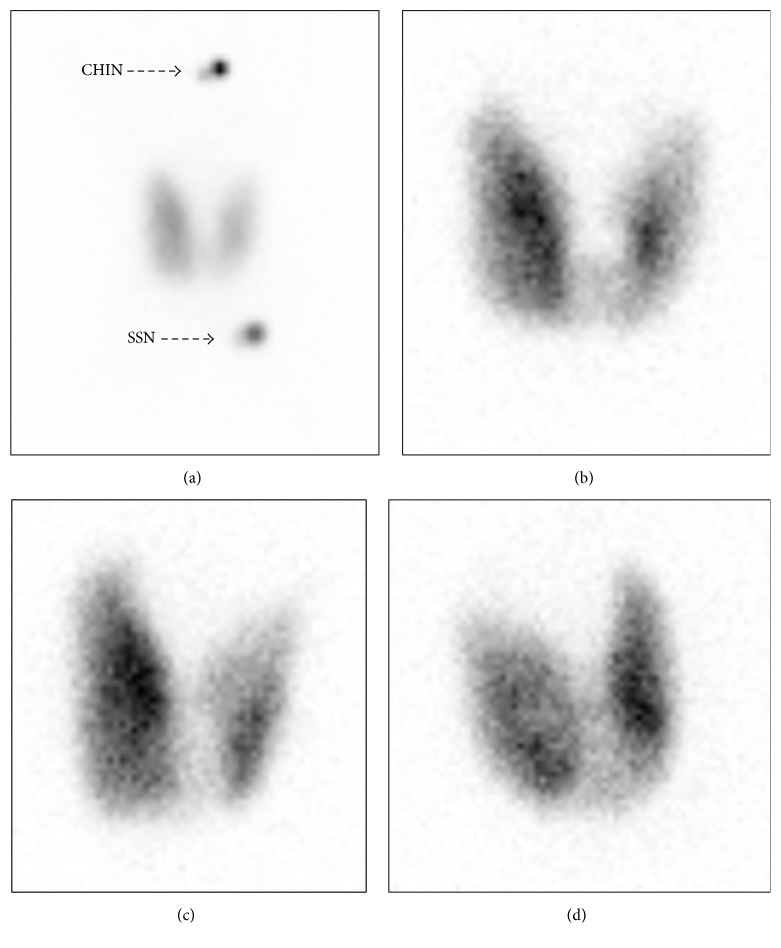
Twenty-four-hour radioactive iodine-123 uptake test. The images show diffuse uptake of 45% in the anterior view at high resolution with markers (a), anterior view with the pinhole collimator (b), right anterior oblique view with the pinhole collimator (c), and left anterior oblique view with the pinhole collimator (d). SSN: substernal notch.

A 76-year-old diabetic man presented to an outpatient clinic with fatigue. He was found to have a total thyroxine (T4) of 22.5 *μ*g/dL (normal 4.5–12.1 *μ*g/dL) and an undetectable thyroid stimulating hormone (TSH) (normal 0.358–3.740 IUI/mL). Diagnostic studies for hyperthyroidism found a thyroglobulin antibody of <20 IU/mL (normal < 40 IU/mL), thyroid peroxidase antibody of 17.0 IU/mL (normal < 35 IU/mL), thyroid stimulating immunoglobulin of 490% (normal ≤ 122%), thyroid stimulating hormone receptor antibody of 3.91 IU/L (normal ≤ 1.75 IU/L), 24-hour radioactive iodine-123 uptake of 45% (normal 10–30%) (Figure 1), and multiple smaller-than-one-centimeter thyroid nodules via an ultrasound of the thyroid. Physical examination did not find ophthalmopathy or dermopathy. He was diagnosed with Graves' disease for which he was started on methimazole 10 mg daily. Over the next two months, he developed dyspnea on exertion, insomnia, fevers, night sweats, productive cough, and weight loss. He presented back to the clinic in atrial fibrillation with rapid ventricular rate and was admitted to the hospital.

**Figure 2:**
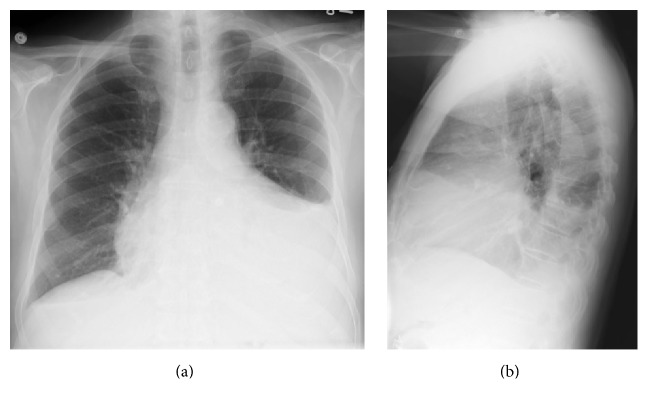
Conventional chest radiograph on the day of admission. The images show an enlarged cardiac silhouette, bibasilar opacities, and left-sided pleural effusion.

On the day of admission, conventional chest radiography found an enlarged cardiac silhouette, bibasilar opacities, and left-sided pleural effusion (Figure 2). Cardiac markers included N-terminal prohormone of brain natriuretic peptide of 1,172 pg/mL (normal 0–450 pg/mL) and two-consecutive troponin I < 0.017 ng/mL (normal 0–0.059 ng/mL). Thyroid hormone levels were TSH of 0.007 UIU/mL (normal 0.358–3.740 IUI/mL), free T4 3.7 ng/dL (normal 0.7–1.6 ng/dL), total T4 21.3 *μ*g/dL (normal 4.5–12.1 *μ*g/dL), and free triiodothyronine 235 ng/dL (normal 55–172 ng/dL). Electrocardiography showed diffuse low voltage and was consistent with atrial fibrillation with rapid ventricular rate. Methimazole was increased to 30 mg daily. He was started on continuous diltiazem infusion (the patient had an allergy to beta blockers), continuous heparin infusion for atrial fibrillation, and ceftriaxone and azithromycin for possible community-acquired pneumonia.

**Figure 3:**
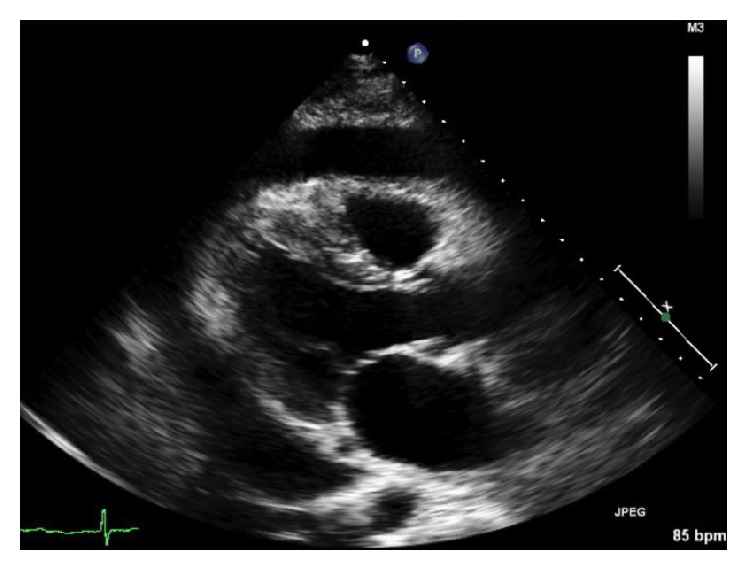
Transthoracic echocardiography (TTE) performed on day one of the hospitalization. The image shows a pericardial effusion.

On hospital day 1, transthoracic echocardiography (TTE) found a pericardial effusion (Figure 3), mitral wave flow variation of greater than 30%, mild right ventricular diastolic compression, and plethoric inferior vena cava with minimal reactivity. With continuous heparin infusion being discontinued shortly before the procedure, pericardiocentesis with the placement of a pericardial drain drained 850 mL of sanguineous fluid during the procedure, and 200 mL of fluid was drained during the subsequent day. Studies of the pericardial fluid found 607,000 red blood cells (RBCs), 4,548 total nucleated cells, 49% neutrophils, 15% lymphocytes, 36% mononuclear cells, 0% eosinophils, 0% other cells, 0% nucleated cells, glucose of 121 mg/dL (no reference range), lactate dehydrogenase (LDH) of 1,326 units/L (no reference range), specific gravity of 1.030 (no reference range), and total protein of 4.8 g/dL (no reference range). On that subsequent day, continuous diltiazem infusion was transitioned to oral diltiazem and he began aspirin.

On hospital day 3, he experienced worsening dyspnea, fever, and diarrhea consistent with thyroid storm, for which he received propylthiouracil potassium iodide, hydrocortisone, and cholestyramine. A left-sided thoracentesis was performed for increasing dyspnea and oxygen requirement and drained 1,250 mL of serous fluid. Studies of the pleural fluid found 1,000 RBCs, 364 total nucleated cells, 30% neutrophils, 44% lymphocytes, 26% mononuclear cells, 0% eosinophils, 0% other cells, 0% nucleated red blood cells, glucose of 140 mg/dL (no reference range), LDH of 78 units/L (no reference range), pH of 7.432 (no reference range), and total protein of 2.6 g/dL (no reference range). Studies were consistent with transudative effusion by Light's criteria. On hospital day 5, the pericardial drain was removed, and TTE two days later showed trivial pericardial effusion. Cultures of the blood, pericardial fluid, pleural fluid, and sputum had no growth. Cytology of the pericardial fluid did not find malignant cells. He converted spontaneously to sinus rhythm and was discharged on methimazole 30 mg orally daily, hydrocortisone 15 mg orally every morning and 10 mg orally every evening, diltiazem 240 mg orally daily, and aspirin 81 mg orally daily. At follow-up clinic visits six weeks and five months later, he was asymptomatic, free T4 was normal, and ECG revealed sinus rhythm and normalization of voltage.

## 3. Discussion

Pericardial effusions occur in approximately 3% to 6% of patients with hypothyroidism [9]. Contrastingly, in our review of the literature, we found only 10 previously reported cases of pericardial effusion in the setting of hyperthyroidism (Table 1). Four of the pericardial effusions were sanguineous [2, 5, 7, 8]. Similar to our patient, six of the patients had Graves' disease [2, 3, 7, 8]. Five of the 10 reported patients had atrial fibrillation [2, 6–8]. Our patient's constellation of sanguineous pericardial effusion and atrial fibrillation treated with continuous heparin infusion appears unique among the available case reports, and the heparin may have contributed to the blood in our patient's pericardial effusion.

The mechanism for the development of a pericardial effusion with Graves' disease has not been elucidated. Previous authors have postulated that the mechanism is similar to that of the ophthalmopathy and myxedema found in hyperthyroidism [2, 5, 8]. A study of hypothyroidism found shifts in extravascular and intravascular proteins and a decrease in lymph drainage [10]. Hyperthyroidism may involve a similar pathophysiology. Given the limited information provided in the available case reports, the contribution of anticoagulation to the sanguineous nature of the pericardial effusion is unknown. For our patient, the presence of RBCs in the pericardial effusion and pleural effusion, albeit a small amount in the pleural effusion, suggested that anticoagulation may be a factor in the development of sanguineous pericardial effusions in the setting of Graves' disease, assuming that the procedures were not notably traumatic.

In the case of pericardial effusions, the absence or presence of tamponade physiology can dictate the necessity of urgent pericardiocentesis. Our patient developed cardiac tamponade physiology and required pericardiocentesis. In several of the previously reported cases, the pericardial effusion resolved with treatment of hyperthyroidism alone [2, 3, 6].

Pericardial effusions usually undergo further diagnostic evaluation, as the etiology may be caused by malignancy or tuberculosis. In a United States study, 64% of pericardial effusions were sanguineous, and malignancy and tuberculosis caused 26% and 1%, respectively, of the sanguineous pericardial effusions [11]. Seventy percent of malignant pericardial effusions were serosanguineous or sanguineous in an Israeli population [12]. In other studies of sanguineous pericardial effusions, malignancy and tuberculosis accounted for 13–45.6% and 4–28.6%, respectively, of pericardial effusions [13–16]. Our patient appeared to have neither of these etiologies, as culture and cytology of his pericardial fluid did not grow mycobacteria or reveal malignant cells. Furthermore, with treatment for his hyperthyroidism, his pericardial effusion did not recur.

We describe a case of a patient with Graves' disease complicated by sanguineous pericardial effusion, cardiac tamponade, and atrial fibrillation. This condition is rare, with only 10 previously reported cases. Our case is similar to previously reported cases, except that we believe this is the first reported case of sanguineous pericardial effusion involving a heparin infusion. Clinicians should be mindful that although rare, pericardial effusions and cardiac tamponade can be due to hyperthyroidism and that sanguineous pericardial effusion, usually associated with malignancy or tuberculosis, can be due to Graves' disease. Because a pericardial effusion may be sanguineous, the risk of bleeding from the use of anticoagulation for atrial fibrillation should be considered in the setting of hyperthyroidism. The American Heart Association, the American College of Cardiology, and the Heart Rhythm Society recommend administering anticoagulation in the setting of hyperthyroidism based on validated risk factors and risk scoring systems such as CHA_2_DS_2_-VASc (congestive heart failure, hypertension, age ≥75 years [doubled], diabetes mellitus, prior stroke or transient ischemic attack or thromboembolism, vascular disease, age 65 to 74 years, and sex category) [17, 18].

## Figures and Tables

**Table 1:** Case reports of hyperthyroidism complicated by pericardial effusion.

Reference	Age (years), gender	Etiology of thyroid disease	Presence of tamponade	Presence of atrial fibrillation	Sanguineous pericardial effusion	Cardiac intervention
Clarke et al. [2]	53, male	NS	Yes, by clinical features	NS	Yes	Pericardiocentesis, pericardiectomy
Clarke et al. [2]	35, female	Graves' disease	Yes, by clinical features	NS	NS	Pericardiocentesis
Clarke et al. [2]	54, female	NS	No, by ECHO	Yes	Described as “unremarkable”	Pericardial biopsy
Clarke et al. [2]	47, female	Graves' disease	No, by ECHO	Yes	NS	None
Khalid et al. [3]	68, female	Graves' disease	No, by ECHO	NS	NS	None
Levy et al. [4]	NS, NS	NS	NS	NS	NS	NS
Nakata et al. [5]	43, male	Graves' disease	No, by ECHO	No	Yes	Pericardiocentesis
Ovadia et al. [6]	76, female	Multinodular goiter	No, by ECHO	Yes	NS	None
Teague et al. [7]	42, female	Graves' disease	Yes, by ECHO	Yes	Yes	Pericardiocentesis
Yu et al. [8]	33, female	Graves' disease	Yes, by ECHO	Yes	Yes	Pericardiocentesis
Current case	76, male	Graves' disease	Yes, by ECHO	Yes	Yes	Pericardiocentesis

ECHO: echocardiography; NS: not stated in case report.

## Abbreviations

ECG: Electrocardiography
LDH: Lactate dehydrogenase
RBC: Red blood cell
TSH: Thyroid stimulating hormone
T4: Thyroxine
TTE: Transthoracic echocardiography.
